# Psychophysiological Response Patterns to Affective Film Stimuli

**DOI:** 10.1371/journal.pone.0062661

**Published:** 2013-04-30

**Authors:** Marieke G. N. Bos, Pia Jentgens, Tom Beckers, Merel Kindt

**Affiliations:** 1 Department of Clinical Psychology, University of Amsterdam, Amsterdam, The Netherlands; 2 Cognitive Science Center Amsterdam, University of Amsterdam, Amsterdam, The Netherlands; 3 Department of Cognitive Psychology, VU University Amsterdam, Amsterdam, The Netherlands; 4 Department of Vision and Cognition, Netherlands Institute for Neuroscience, an Institute of the Royal Netherlands Academy of Arts and Sciences, Amsterdam, The Netherlands; 5 Department of Psychology, KU Leuven, Leuven, Belgium; University of Jyväskylä, Finland

## Abstract

Psychophysiological research on emotion utilizes various physiological response measures to index activation of the defense system. Here we tested 1) whether acoustic startle reflex (ASR), skin conductance response (SCR) and heart rate (HR) elicited by highly arousing stimuli specifically reflect a defensive state and 2) the relation between resting heart rate variability (HRV) and affective responding. In a within-subject design, participants viewed film clips with a positive, negative and neutral content. In contrast to SCR and HR, we show that ASR differentiated between negative, neutral and positive states and can therefore be considered as a reliable index of activation of the defense system. Furthermore, resting HRV was associated with affect-modulated characteristics of ASR, but not with SCR or HR. Interestingly, individuals with low-HRV showed less differentiation in ASR between affective states. We discuss the important value of ASR in psychophysiological research on emotion and speculate on HRV as a potential biological marker for demarcating adaptive from maladaptive responding.

## Introduction

Humans are endowed with a wide repertoire of emotions, which are essential to signal changes in environmental demands to facilitate adaptive coping strategies and action tendencies [1], [2]. Emotional responses can be organized along the dimensions of motivation (defensive-appetitive), arousal and hedonic valence [3] and consist of a complex interplay between multiple response systems like cognition, behavior and physiology [1], [4]–[6]. Experimental studies on emotional response patterns contribute to our understanding of the adaptive and maladaptive functions of the motivational system [7]–[11]. Given that the majority of these studies use psychophysiological measures to assess emotional responses, it is important to unravel the specificity of these physiological measures.

Here, we examined the effects of exposure to short affective film clips (i.e., negative, positive, neutral) on startle reflex, skin conductance and heart rate. Psychophysiological studies on affective states often assess only a single physiological response measure to index activation of the defense system. The first aim of the study was to investigate whether these physiological indices could differentiate between positive and negative valence and thus represent activation of the defense system specifically rather than activation of the motivational system (arousal) in general. Interpretation of a physiological response as a defensive response is only appropriate if the physiological index shows diverging response patterns in reaction to positive, neutral and negative stimuli.

The acoustic startle reflex (ASR) is strongly modulated by the valence of background stimuli [3], [12], [13]. The ASR can be evoked by a loud noise and is characterized by an integrative, reflex contraction of the skeletal musculature. Its pathway is directly connected with the amygdala [14], [15], which is considered a prominent structure of the defense network [5], [16]. The amygdala modulates processing of affective stimuli such that both aversive and pleasant affective states can influence the ASR [3], [13]. In contrast to ASR, SCR is supposed to primarily reflect autonomic arousal, regardless of whether the stimulus induces a negative or positive state [3], [17], [18]. Changes in SCR represent activity within the sympathetic axis of the autonomic nervous system (ANS), measured by autonomic innervation of the sweat glands at the surface of the skin [17], [19]. The neurobiological underpinnings of SCR are widespread and not exclusively related to the defense network [17]. Likewise, heart rate (HR) is also under control of the ANS [20]. Deceleration of HR is often shown in response to affective stimuli and most pronounced to negative stimuli [7], [21], [22]. HR deceleration seems to be related to more attentional processing regarding changes in affective states [3] rather than valence or arousal. In sum, ASR, SCR and HR seem to represent partly different neural systems.

Film clips are among the most powerful stimuli to elicit affective responses in an experimental lab setting [23], [24]. Advantages of film stimuli in comparison to other stimuli are their dynamic nature and the fact that they simultaneously provide visual and auditory input, which makes the presentation of film clips an ecologically valid methodology to induce different affective states [25], [26]. Another advantage is that film clips may induce a more sustained affective state compared to presentation of pictures like the IAPS that elicit only short-lived affective responses. Previous studies that used the film-viewing paradigm to investigate physiological response patterns of positive and negative affective states mainly focused on physiological response patterns within the ANS (e.g., SCR, HR) [7], [21], [22], [27] or specifically on the affective modulation of the startle reflex [12], [28], [29]. However, to unravel the differences between ASR, SCR and HR, these physiological indices should be assessed simultaneously. So, in order to observe differences in physiological response patterns, we used a *within-*subject design to manipulate different affective states within an individual while ASR, SCR and HR were obtained concurrently.

Given that individuals vary in their ability to differentiate and regulate affective states, the second aim of our study was to investigate individual differences in physiological response patterns. Heart rate variability (HRV) has recently received much attention as a potential biological marker of individual differences in affective responding. The heart is dually innervated by both the sympathetic and the parasympathetic nervous system, with activation of the former resulting in a relative increase and activation of the latter in a relative decrease in heart rate. HRV reflects the continuous interplay between both nervous systems and is regarded as a measure of autonomic flexibility and even as a biological marker of emotional responding [30]–[32]. Lowered HRV has for example been linked to various psychological disorders, such as anxiety disorders and depression [33]–[35]. Interestingly, HRV seem also to be associated with differences in affective modulation of the ASR. It has been shown that low resting HRV was related to less affective modulation of the ASR in a picture-viewing paradigm [36] and in a threat-of-shock procedure [11]. The observed relation between resting HRV and startle modulation may be explained by the neurobiological substrates of both indices. It has been proposed that HRV reflects the level of inhibitory control of the prefrontal cortex, not only over sympathoexcitatory circuits but also over defensive networks [32], [37]. Hence, both ASR and HRV seem to be related to activation of the defense system. To add to the aforementioned studies, here we examined the relation between resting HRV and response patterns to sustained affective states. Thereby, the current study may contribute to the sparse literature on the relation between resting HRV and ASR. Furthermore, we explored whether resting HRV is also related to physiological indices that may not directly be linked to the defense system, like SCR and HR.

In the present experiment we used a film-viewing paradigm in which participants were exposed to negative, positive and neutral film stimuli. ASR was elicited by a sudden loud noise at the start and the end of each film clip. HR and skin conductance were continuously recorded during the experiment. HRV was assessed during a 5-minute baseline period prior to the presentation of the film clips. Based on previous studies, we hypothesized that ASR would be enhanced during the negative film clips and reduced during the positive film clips compared to the neutral film clips [3], [12], [13], [28]. In contrast, SCR was expected to respond to highly arousing stimuli regardless of whether they are positive or negative [3], [27]. Furthermore, we expected a deceleration of HR during all stimuli, but more markedly during negative film clips [7], [21], [27]. With respect to individual differences, we hypothesized that HRV would be associated with ASR modulation [36] and we further explored whether HRV would also be related to SCR and HR.

## Methods

### Ethics Statement

The ethical committee of the University of Amsterdam approved the study (reference number 2010-KP-1005). Written informed consent was obtained from all participants.

### Participants

Thirty-five healthy students participated in the study, ranging in age between 18 and 25 years (*M = *20.6 years; 12 men). Participants received either course credits or were paid a small amount of money (€10) for their participation.

### Film Stimuli

Twelve film clips were presented that were intended to elicit a negative, a positive or a neutral state [25], [26]. The film clips varied in duration from 55 to 77 s, with an average duration of 65 s. Small variations in duration of the film clips were accepted in order to allow for clear and sensible content of the clips and build-up to a climax. The sound of the film clips was presented binaurally at a medium volume (45–55 dB) through headphones (Sennheiser 25-I II). A set of film clips was used for each affective state to ensure that reactions were related to the induced affective state by the film clips and not to the specific contents of particular clips [26]. The negative film clips were scenes from *Cujo* (1983; Lewis Teague), *Silence of the Lambs* (1991; Jonathan Demme), *Irreversible* (2002; Gaspar Noé) and *History X* (1998; Tony Kaye). The negative film clips that we used represent fear-provoking themes of anticipated or immediate threat and bodily injury. The positive clips were two erotic scenes from *Meet Joe Black* (1998; Martin Brest) and *Summerheat* (2008; Monique van de Ven) and two sport clips from the soccer World Cup Match (1998; The Netherlands versus Argentina) and the Olympic Games (1992; race of the Dutch athlete Ellen van Lange). We selected two categories of positive clips because erotic scenes - although often used as positive and arousing stimuli - not always induce a distinctly positive affective state [12]. The neutral film clips were selections from documentaries and depicted little activity and bland outdoor scenes.

### Measurements

#### Startle probe

The startle probe was a 40-ms burst of 104-dB white noise with near-instantaneous rise time. Two startle probes were presented during each film clip. The first probe was presented between 15 and 25 s after film onset (M = 19 s), the second probe was presented during the climax of each film clip between 51 and 75 s (M = 61 s). The scheme of startle presentations was identical for all participants. The startle probes were presented binaurally over the headphones.

#### Anxiety assessment

Individual anxiety levels were obtained in order to relate these levels to individual differences in physiological responding. State and trait anxiety were assessed with the State and Trait Anxiety Inventory [38]. The Anxiety Sensitivity Index [39] was used to measure participants’ tendency to respond fearfully to anxiety-related symptoms.

#### Retrospective ratings of the film clips

To ensure that the film clips elicited the intended affective states, participants rated their emotional experience to the film immediately after each film clip using the Self-Assessment Manikin [40] and the Positive and Negative Affect Schedule [41]. The SAM is a nonverbal self-report measure composed of three series of five pictograms, depicting increasing levels of valence, arousal and dominance, respectively. The PANAS is a self-report measure of positive and negative affect. It contains 20 emotion-related words; the participants had to rate to what extent they experienced the state corresponding to each of these words as they were watching the film clip.

#### Physiological recording

Potentiation of the acoustic startle reflex to a loud noise was measured by electromyography (EMG) of the right orbicularis oculi muscle. 7-mm Ag/AgCl electrodes filled with electrolyte gel were attached approximately 1 cm under the pupil and 1 cm below the lateral canthus, respectively; a ground electrode was placed on the forehead [42]. The EMG electrode wires were connected to a front-end amplifier with an input resistance of 10 MΩ and a bandwidth of DC-1500 Hz. The signal was digitized at 1000 S/s. Electrodermal activity was measured using an input device with a peak-peak sine shaped excitation voltage (±0.5 V) of 50 Hz. The input device was connected to two Ag/AgCl electrodes of 20 by 16 mm, which were attached to the medial phalanx surfaces of the middle and fourth finger of the non-dominant hand. The signal from the input device was led through a signal-conditioning amplifier and the analog output was digitized at 1000 Hz by a 16-bit AD-converter (National Instruments, NI-6224). Beat-to-beat heart rate (HR) was measured by electrocardiography (ECG). ECG was recorded from three AgAg/CL electrodes, attached via the modified lead-2 placement and digitized at 1000 Hz.

### Experimental Design and Procedure

All participants were tested individually in a sound-attenuated room. Before the experiment started, participants filled in three questionnaires: ASI, trait and state anxiety (STAI). During the experiment, participants sat in a comfortable chair at a distance of 70 cm from a computer monitor. After attachment of the ECG, EMG and skin conductance electrodes participants were informed about the procedure. During the first 5 min ECG was recorded for HRV analysis, while participants listened to relaxing music (Frank Borell’s *“Landpartie”*, 2007). Thereafter, participants were instructed to carefully watch each film clip and to rate the feelings they experienced during the film immediately afterwards on the computer screen. The task started with a habituation phase containing ten acoustic startle probes to stabilize baseline startle reactivity, followed by the presentation of the twelve film clips. Between the online questionnaires and the next film clip a short inter-trial interval was inserted of 20 s (range: 15–25 s) containing a black screen and fixation cross. Film clips were presented semi-randomly, with the restriction that no more than two consecutive trials were of the same type. The first trial always contained a neutral film clip (Note: Due to failures in randomization of the first three film clips, a neutral film clip was always followed by a negative film clip and thereafter a positive (erotic or sport) film clip. Analyses that excluded the first three film clips yielded similar results as the analyses presented below).

### Data Reduction and Response Definition

All physiological data were processed with VSRRP 98 v 8.0 (developed by Technical Support Group, UvA Psychology). For the startle response data, an analog notch filter was set at 50 Hz to remove interference of the mains noise. The raw EMG signal was amplified and band-pass filtered (28–500 Hz butterworth 4^th^ order) [42], [43]. Startle magnitude was defined as the amplitude (measurement unit: µV) of the first peak within a 20–200 ms interval following the startle probe onset. Trials with excessive baseline activity or recording artifacts were discarded. To reduce inter-individual differences raw scores were z-transformed and converted to T-scores (T = (z×10) +50).

Skin conductance was measured continuously during the film clips. In order to compare the physiological measurements and to avoid response artifacts from the startle probes, statistical analyses were performed over the average skin conductance response (SCR, measurement unit: µS) over a period of 10 s *prior* to the first and second startle probes relative to baseline. The baseline was taken 5 s before each film clip. Interference of the first startle probe during the second response window of SCR was minimized by a relatively long time interval between the startle probes (*M = *42 s; range 29–55 s). Given that the temporal interval between peak and point of 50% recovery of SCR amplitude is between 2–10 s [19], we do not expect SCR during the second time interval to be influenced by the first startle probe. Like the startle data, SCRs were z-transformed and converted to T-scores.

The ECG beat-to-beat data were visually screened for physiologically impossible readings and artifacts and hand-corrected. Heart rate in beats per minute (BPM) during film clips was calculated with VSRRP. Statistical analyses were performed on HR scores over a period of 10 s prior to the startle probes minus baseline, which was taken 5 s before film onset. For HRV, we used the ECG data of the 5 min baseline period, which was taken before the start of the presentation of the film clips. The high-frequency (HF) component of HRV was used as our estimate of vagally-mediated HRV. HF-HRV was obtained with Kubios HRV Package, developed by the Biosignal Analysis and Medical Imaging Group, Department of Physics, University of Kuopio, Finland. IBIs were imported to KUBIOS and artifact-corrected (with low artifact rejection and smooth prior correction). HF-HRV was derived with a standard Fast Fourier Transformation and using a high frequency band from 0.15–0.40 Hz. To normalize the distribution of the HF component scores we log-transformed the data. In the current study we did not control for respiration influences; uncorrected measures of HRV have been reported to be at least as good an index of vagally-mediated cardiac control as those corrected for respiration parameters [44]. One participant was identified as an outlier on logHF-HRV and was excluded from HRV analyses [45].

### Statistical Analysis

*Preliminary analyses* were performed to test whether the four selected film clips *within* each affective category (i.e., negative, positive, neutral) did not differ from each other on valence and arousal ratings. The SAM ratings were analyzed with repeated-measures analysis of variance (ANOVA). Paired-samples t-tests were performed to further assess differences between the film clips within each affective category, when the omnibus test was significant. Thereafter, we performed a *manipulation check* to test for univariate differences in subjective ratings *between* the affective categories. SAM ratings and PANAS scores were analyzed with repeated-measures ANOVAs with Affect as within-subject factor.

To test the effects of exposure to affective film categories on ASR, SCR and HR, we used separate repeated measures ANOVAs with again Affect as within-subject factor. Significant effects were followed-up by contrast analyses and t-test (two-tailed). For the contrast analyses, we ordered the film categories from positive (sport, erotic), neutral to negative. In this way, a linear contrast suggests a relationship with valence, whereas a quadratic contrast indicates a relation with arousal. Finally, to investigate the effect of HRV on affective responding, we divided the participants in groups of low and high HRV based on logHF-HRV at rest using median split [45]. We performed mixed repeated-measures ANOVA with HRV-group as between subject factor and Affect as within-subjects factor for subjective ratings as well as the physiological indices. Additionally, we calculated correlations between HRV, ASR, SCR, HR and anxiety ratings.

For the ANOVAs, a Greenhouse-Geisser procedure was applied when the assumption of sphericity was violated and the uncorrected degrees of freedom and the epsilon are then reported. The alpha level of.05 was used for all statistical analyses. In case of multiple comparisons at follow-up analyses, Bonferroni correction was used to control for false positives (i.e., *p* = 0.05/6 [number of tests]) = 0.008).

## Results

### Preliminary Analysis

The four neutral film clips did not differ in the valence and arousal ratings of the SAM (*Fs*<1.9). For the positive film clips, there was a difference in valence (*F*(3,102) = 7.39, *p*<0.01, η_p_^2^ = .18, ε = .67), but not in arousal ratings (*F*<1.5). Follow-up analyses revealed no difference in valence ratings between the two sport or the two erotic film clips (*Fs*<1.0), but did yield a difference between these two positive categories (*F*(1,34) = 12.36, *p*<0.01, *η_p_^2^* = .27). The negative film clips were differentially rated on the valence scale (*F*(3,102) = 18.12, *p*<0.0001, *η_p_^2^* = .35, ε = .78). Pair-wise comparisons indicated that the scene taken from History X was rated as more aversive than the other three clips (*ts*_34_>3.30, *p*s<0.008, *ds>*.56). Furthermore, the film clip taken from Irreversible was rated slightly more negative than the film clips from Cujo and Silence of the Lambs (*ts*_34_>2.69, *ps*<0.011, *ds*>.45). Ratings on the arousal scale also indicated a difference between the negative film clips (*F*(3,102) = 8.55, *p*<0.0001, *η_p_^2^* = .20). For arousal, only History X differed significantly from the other clips (*ts*_34_>3.04, *ps*<0.008, *ds>*.52).

In sum, the preliminary analyses demonstrated a distinction between the erotic and sport clips in valence ratings. Therefore, we split the positive category into two categories in the results below (i.e., sport and erotic film category). In order to match the number of film clips for each film category, we excluded two clips from the negative and neutral category. For the negative category we excluded the film clips from History X and Irreversible, because both differed significantly from the other two clips in valence ratings. Thus, the analyses below were performed over four affective film categories (i.e., negative, positive-sport, positive-erotic and neutral), which all consisted of two film clips.

### Manipulation Check

A first manipulation check was performed to test whether the film clips induced the intended affective state. Participants rated the four affective categories (i.e., negative, positive-sport, positive-erotic, neutral) as significantly different on the valence scale of the SAM (*F*(3,102) = 58.62, *p*<0.0001, *η_p_^2^* = .63, ε = .78). Pair-wise comparisons indicated that all affective categories differed from each other (*t*s_34_>3.51, *p*s<0.008, *d*s*>*0.60). As shown in Table 1, the sport film clips were rated as most positive followed by the erotic, neutral and negative film clips, respectively. Similar results were obtained from the Positive Affect scale of the PANAS (*F*(3,102) = 33.88, *p*<0.0001, *η_p_^2^* = .50, ε = .62). Pair-wise comparisons revealed significant differences between all affective categories (*ts*_34_>4.14, *p*s<0.008, *d*s*>*.72), except negative versus neutral (*t*_34_ = 0.78, *p*>.10). Analysis of the Negative Affect scale of the PANAS also revealed a significant effect of Affect (*F*(3,102) = 36.01, *p*<0.0001, *η_p_^2^* = .51, ε = .47). Follow-up analyses demonstrated that the negative film clips were rated more negative than the erotic, sport and neutral film clips (*ts*_34_>6.22, *p*s<0.008, *d*s*>*1.32). Given that erotic film clips in addition to positive feelings may also induce feelings of shame [12], we additionally examined the negative affect item *shame* of the PANAS. The repeated measures ANOVA yielded a main effect of category (*F*(3,102) = 7.85, *p*<0.001, *η_p_^2^* = .19). Follow-up analyses indicated that the erotic film clips elicited more feelings of shame compared to the sport and neutral clips (*ts*_34_>2.83, *ps<*0.008, *ds>*0.52).

**Table 1:** Means and standard deviations of self-reported emotional experience during the film clips.

	Negative	Neutral	Sport	Erotic
	*M (SD)*	*M (SD)*	*M (SD)*	*M (SD)*
SAM Valence	4.4 *(1.4)*	5.5 *(1.2)*	7.4 *(1.2)*	6.7 *(1.0)*
SAM Arousal	4.7 *(1.7)*	1.7 *(0.9)*	3.2 *(1.7)*	3.2 *(1.6)*
SAM Dominance	4.8 *(1.6)*	5.7 *(1.3)*	6.4 *(1.3)*	5.8 *(1.3)*
PANAS Positive Affect	2.1 *(0.5)*	2.1 *(0.5)*	3.1 *(0.7)*	2.4 *(0.6)*
PANAS Negative Affect	1.8 *(0.6)*	1.1 *(0.2)*	1.1 *(0.2)*	1.2 *(0.2)*

Note: SAM ratings range from 1 to 9 (1 =  unpleasant, low-arousal, low-dominance). PANAS ratings range from 1 to 5 (1 =  “not at all” to 5 =  “extreme”).

A second manipulation check was performed to test whether the negative, erotic and sport film clips induced higher levels of arousal compared to the neutral film clips. Ratings of the SAM arousal scale indicated a significant effect of Affect (*F*(3,102) = 37.71, *p*<0.0001, *η_p_^2^* = .53). Apart from the erotic versus sport category (*t*_34_ = 0.0, *p*>.1), all affective categories differed from each other (*t*s_34_>4.90, *p*s<0.008, *d*s*>*0.83). Thus, the negative film clips were also rated as more arousing than the positive-erotic and positive-sport film clips (see Table 1). This unintended difference between pleasant and unpleasant stimuli is, however, also observed in other studies [10], [46], [47].

Additionally, we examined the dominance ratings of the SAM scale. There was a significant effect of Affect (*F*(2,103) = 15.04, *p*<0.0001, *η_p_^2^* = .31, ε = .68). Except for the neutral versus erotic film clips (*t*_34_ = 0.15, *p*>.10), all affective categories differed from each other (*ts*_34_>3.03, *p*s<0.008, *d*s*>*0.52). Participants felt overwhelmed by the negative film clips and felt rather dominant during sport film clips (Table 1).

Taken together, the manipulation largely succeeded. The four film categories induced the intended affective state. Although the negative, sport and erotic film clips induced more arousal than the neutral film clips, the degree of arousal was stronger during the negative film clips.

### Affective Responding during Film Viewing

#### Startle magnitude

**Figure 1:**
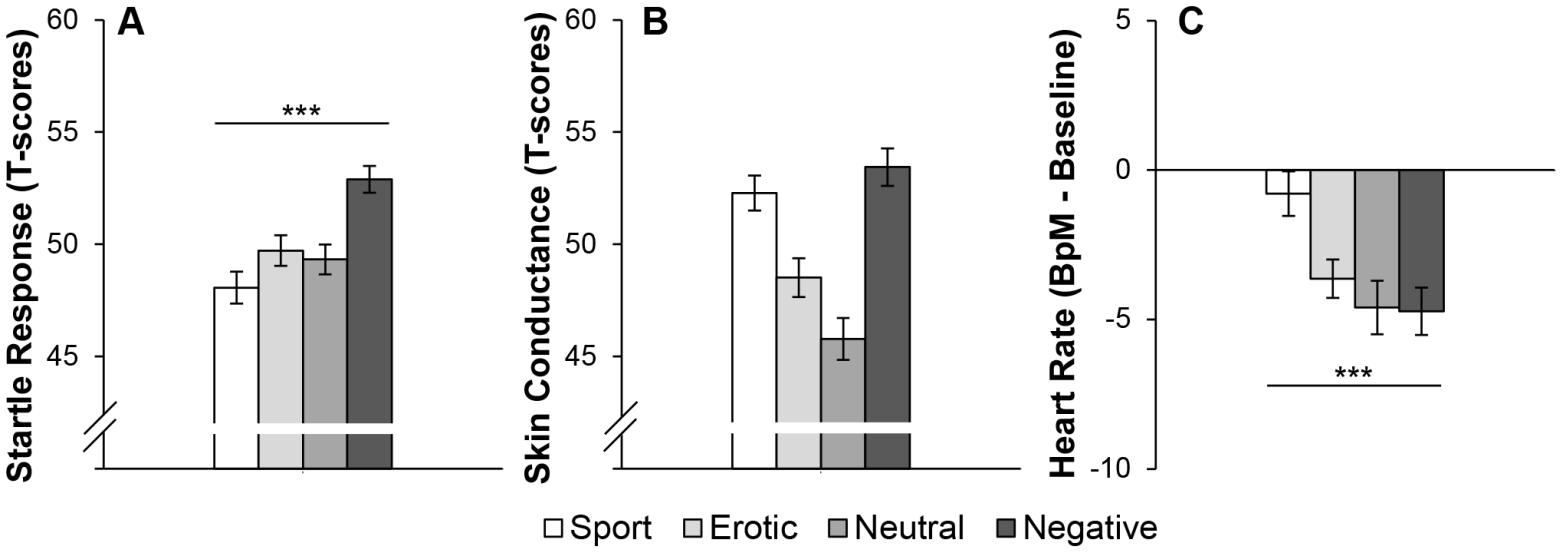
Physiological indices per Affective Category. Panel A presents the average ASR data. Panel B presents the average SCR 10 seconds prior to both startle probes. Panel C presents average HR data relative to baseline of the 10 seconds prior to the startle probes. The film clips are ordered by subjective valence ratings, the line represents the linear trend of valence. ***p<0.001; Error bars represent SEM.

Analysis of the ASR revealed a significant main effect of Affect (*F*(3,102) = 7.20, *p*<0.001, *η_p_^2^* = .18). Contrast analysis confirmed that ASR was modulated by valence (linear contrast: *F*(1,34) = 23.98, *p*<0.001, *η_p_^2^* = .41; quadratic contrast: *F*(1,34)<1.26). As can be seen in Figure 1a, negative film clips elicited the strongest ASR followed by the erotic, neutral and sport film clips. Additionally, pair-wise comparisons confirmed augmented ASR to negative film clips compared to the other film clips (*ts*_34_>3.11, *ps*<0.008, *ds*>.52). Although Figure 1a might suggest startle inhibition to the sport film clips, there was no significant difference between the sport and neutral film clips (*t*_34_<1.10).

#### Skin conductance

Electrodermal responding to the different affective film categories is depicted in Figure 1b. Analysis of SCR showed a main effect of Affect (*F*(3,102) = 12.63, *p*<0.001, *η_p_^2^* = .27). Contrast analysis showed that SCR was related to arousal irrespective of valence (linear contrast: *F*(1,34)<1.0; quadratic contrast (*F*(1,34) = 38.92, *p*<0.001, *η_p_^2^* = .53). Pair-wise comparisons demonstrated that the negative and positive-sport film clips differed from the neutral film clips (*ts*_34_>4.61, *p*<0.001, *d*>.78), but also compared to the positive-erotic film clips (*ts*_34_>2.57, *p*<0.015, *d*>.43).

#### Heart rate

As shown in Figure 1c, there was a general deceleration of HR during the film clips relative to baseline. HR differed between the affective film categories (*F*(3,102) = 6.17, *p*<0.01, *η_p_^2^* = .15). Contrast analysis showed that HR was more related to valence than to arousal (linear contrast: *F*(1,34) = 16.16, *p*<0.001, *η_p_^2^* = .32; quadratic contrast: *F*(1,34) = 3.44, *p = *0.07). Pair-wise comparisons revealed that the positive-sport category elicited less HR deceleration than the neutral, negative and erotic film categories (*ts*_34_>3.09, *ps*<0.008, *ds>*.53). We did not observe the expected differentiation between the negative, neutral and erotic film clips (*ts*_34_<1.1).

To summarize, both ASR and HR seem to be related to hedonic valence. Yet, only ASR is primarily affected by a negative state and indicates activation of the defense system. As expected, SCR increased during arousing stimuli regardless of whether the stimuli induced a negative or positive state.

### Resting HRV as a marker for Individual Differences in ASR

#### Median split analysis of resting HRV

The sample was divided in a low and high HRV group based on the median of logHF-HRV (LogHF-HRV; MD = 3.1 ms^2^/Hz; main effect of Group, *F*(1,32) = 41.90, *p*<0.001, *η_p_^2^* = .57). There were no differences between groups on subjective ratings of trait anxiety or anxiety sensitivity (*Fs*<1.0) (see Table 2). Gender distribution was unequal between groups, but could not be statistically tested due to small cell sizes (low-HRV group: 9 males/8 females; high-HRV group: 3 males/14 females).

**Table 2:** Means and standard deviations of logHF-HRV, self-report ratings of anxiety and age by HRV groups.

	Low-HRV	High-HRV
	*M (SD)*	*M (SD)*
logHF-HRV**	2.8 *(0.4)*	3.4 *(0.2)*
Anxiety sensitivity	10.6 *(5.9)*	9.9 *(6.9)*
Trait Anxiety	34.8 *(9.7)*	37.6 *(8.8)*
Age	21.6 *(2.3)*	20.9 *(2.0)*

** *p*<0.001, two tailed.

**Figure 2:**
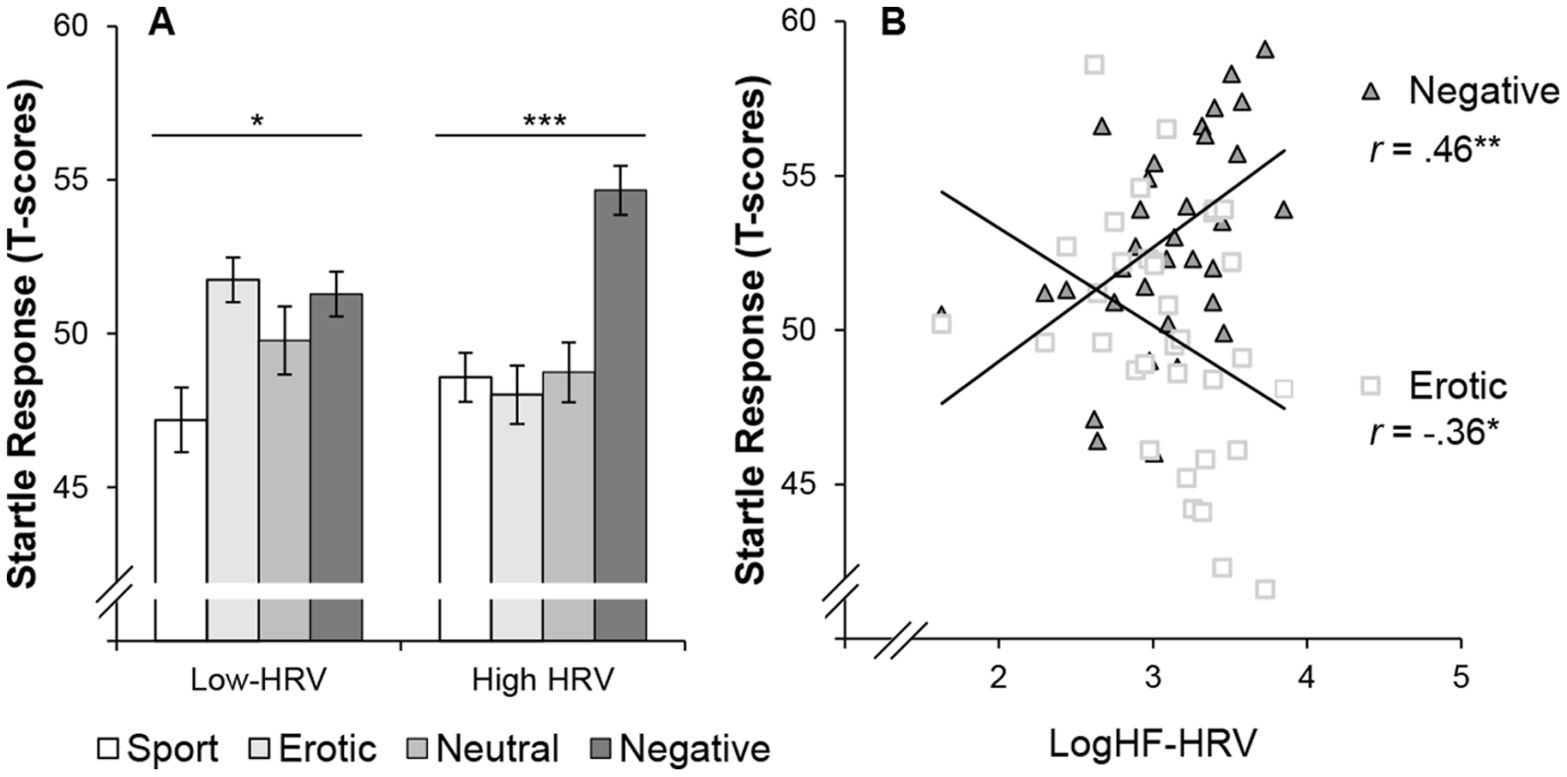
Relation between HRV and startle magnitude. Panel A represents the average ASR elicited by the four affective categories of both HRV groups. The line reflects the main effect of valence. Panel B showed the correlation between logHF-HRV and ASR elicited by the erotic film clips and the negative film clips. ***p<0.001; **p<0.01; *p<0.05; Error bars represent SEM.

The low and high HRV group differed on the affective modulation of the startle reflex (Affect x HRV-group: *F*(3,96) = 4.35, *p*<0.01, *η_p_^2^* = .12). A follow-up analysis showed that both groups showed differentiation in ASR between affective categories (high-HRV group: Affect, *F*(3,48) = 9.33, *p*<0.0001, *η_p_^2^* = .37; low-HRV group: Affect, *F*(3,48) = 3.73, *p*<0.05, *η_p_^2^* = .19). As can be seen in Figure 2a, the high-HRV group showed enhanced ASR during the negative film clips compared to all other film clips (*ts<*3.80, *ps*<0.008, *ds*>0.92). In the low-HRV group, ASR elicited by the negative film clips only differed from ASR potentiation during sport film clips (*t*_16_ = 2.95, *p* = 0.009, *d* = 0.72), but not from the neutral or erotic film clips (*ts<*1.0, *p*>0.1). Also, the erotic film clips elicited stronger ASR than the sport film clips in the low-HRV group (*t*_16_ = 3.08, *p*<0.008, *d* = 0.75). Interestingly, we did not observe any differences between HRV groups in affective responding for SCR (*F*(3,96) = 1.51, *p*>0.1), HR (*F*(3,96) = 1.75, *p*>0.1) or subjective ratings (*Fs*<2.40, *p*>1.0).

To conclude, median split analyses demonstrated that HRV was merely associated with ASR. The affect-modulated characteristics of the ASR were most pronounced in the high-HRV group.

#### Correlations

To provide additional support for the median-split analyses, we calculated correlations between logHF-HRV, ASR, SCR and anxiety measures (i.e., STAI-T, ASI). There was a negative correlation between logHF-HRV and ASR elicited by the erotic film clips (*r* = -.36, *p*<0.05, see Figure 2b) and a positive correlation between logHF-HRV and ASR prompted by the negative film clips (*r* = .46, *p*<0.01, see Figure 2b). Furthermore, there was a correlation between logHF-HRV and HR during the neutral film clips (*r* = .37, *p*<0.05) and sport film clips (*r* = .34, *p*<0.05). There were no significant correlations between logHF-HRV and other physiological indices or anxiety ratings (*rs<*.28, *ps*>0.1).

## Discussion

The present study examined the psychophysiological response patterns to positive and negative affective states elicited by short film clips. The aim of the study was twofold: 1) to test the sensitivity of ASR, SCR and HR to valence and more specifically to a negative, defensive state and 2) to investigate whether vagally-mediated HRV was associated with affective responding. ASR and HR were both related to valence, but only ASR indicated activation of the defense system. In contrast, SCR was associated with arousal, irrespective of the valence of the induced affective state. Furthermore, resting HRV was merely related to the affect-modulated characteristics of ASR. These findings provide valuable insights into the underlying mechanisms of physiological response systems to different affective states and the nature of individual differences in affective responding.

The negative film clips elicited a physiological response cascade that reflects activation of the defense system. These physiological responses are mobilized by different underlying mechanisms, which are all part of the defense system. An isolated physiological response can however not necessarily be interpreted as a defensive response. Consistent with previous work, our data confirm that only ASR can be considered as a reliable and robust indicator of the defense system [3], [12], [14]. Indeed, ASR differentiated between negative, neutral and positive states. The lack of startle inhibition to sexually arousing stimuli is consistent with an earlier study [12], but not with some other studies [3], [48]. Methodological differences between these studies, such as the nature of the stimuli (films versus pictures), may account for the discrepancy in results. It is plausible that erotic film stimuli, as used in the current study and in the study of Jansen and Frijda [12], are interpreted more ambiguously than pictures. Erotic stimuli may not only elicit positive feelings, but can also induce negative emotions as shame and anger [12]. Indeed, further inspection of our data on subjective ratings revealed that the erotic film clips evoked positive feelings as well as feelings of shame.

In sharp contrast to the ASR, SCR increased during both negative and sport film clips. Hence, SCR reflects the level of sympathetic arousal of stimuli rather than a defensive state [3], [17]. The subjective ratings of arousal, however, did not correspond fully with the SCR data, given that the sport and erotic film clips were both rated as highly arousing but only the sport clips elicited a strong SCR. The differentiation between physiology and subjective ratings may be explained by differences in measurement time (i.e., online versus retrospective).

Like SCR, HR does not directly reflect activation of the defense system. In line with previous studies, HR deceleration was modulated by valence [21], [49], although this effect was driven by less HR deceleration during the sport film clips. The failure to observe HR acceleration during negative film clips is a common observation in studies where healthy participants are passively exposed to aversive stimuli [3], [21], [22] and may indicate that mobilization of the defense system is only partial when there is no need for immediate action [5]. Yet, HR acceleration has been shown in phobic patients who were passively exposed to their feared objects [9], [50], [51].

Critically, when we zoomed in on individual differences in affective responding we observed a relation between affect-modulated characteristics of ASR and vagally-mediated HRV. In line with a previous study, we found that individuals in the low-HRV group showed relatively less differentiation in ASR between affective states [36]. Moreover, low-HRV individuals showed a tendency of enhanced ASR to erotic film clips, but not to neutral or sport film clips. So, we found augmented ASR elicited by erotic stimuli rather than a general enhancement of ASR. This result was supported by the correlational analyses. The fact that we did not observe a general enhancement of ASR seem to contradict the results of Ruiz-Padial et al. [36], but may be due to differences in experimental set-up. The sample size as well as the range in HRV scores was smaller in our study. Given that the reported correlations of the aforementioned study were only small to moderate, a lack of power of the current study may explain this discrepancy. In addition, exposure time (i.e., few seconds versus approximately a minute) and stimulus material (i.e., pictures versus film clips) may account for the differences between studies as well. In the current study we examined the relation between resting HRV and physiological response patterns to sustained affective states rather than more initial response patterns induced by picture presentations.

It has been proposed that low resting HRV is associated to hypervigilance for novel and potential threatening stimuli in the environment [36], [52]. Our findings suggest that such hypervigilance may be specific for stimuli that are susceptible to ambiguous interpretations. Although there were no differences in subjective ratings of the affective stimuli between our HRV-groups, the enhanced ASR during the erotic film clips in the low-HRV group may be interpreted as a tendency to react defensively to ambiguous stimuli. Note that the HRV-groups did not differ in anxiety sensitivity or trait anxiety. Remarkably, the high-HRV group did not show the expected startle inhibition to positive film stimuli. It remains unclear how this effect can be explained, given that the subjective ratings confirmed that at least the sport film clips induced a highly positive state. Interestingly, resting HRV was only associated to modulation of the ASR and not to SCR and subjective ratings [53]. This result is consistent with the overlapping neural structures of ASR and HRV, both of which are closely coupled to the neural fear network. As already pointed out, potentiation of the ASR primarily reflects activation of the defense system and the neural circuit involved in ASR is critically dependent on the amygdala [14], [54]. The neurovisceral integration model suggests that the neural substrates of HRV are more widespread and are associated with emotional, attentional and autonomic regulation [32], [52]. HRV may index a reciprocal inhibitory cortico-subcortical neural circuit that forms the structural link between emotion, cognition and health-related physiological processes. According to this model, HRV is linked to the ventromedial prefrontal cortex (vmPFC) and the amygdala [37], [55]. It has been suggested that a reciprocally interconnected neural structure allows the vmPFC to exert inhibitory influence on sub-cortical structures that are highly involved in the defense system such as the amygdala. As such, HRV may be considered as an index for the flexibility of inhibitory control of the vmPFC over the defensive network and is therefore closely related to potentiation of the ASR [32], [36], [37]. It is this reciprocal inhibitory circuit that may be essential for adaptive responding to ever-changing environmental demands [55].

Taken together, the current results indicate that startle potentiation primarily reflects activation of the defense system. In contrast, SCR and HR seem to be related to the motivational system, but not specifically to the defense system. SCR represents the level of arousal of an affective state irrespective of whether the state is positive or negative. HR deceleration was affected by valence, but it did not disentangle a negative state from a neutral or a positive (cf. erotic) state. Thus, the current results emphasize that physiological responses reflect different aspects of the motivational system, such as its intensity of activation (arousal level), and the specific emotional context (valence) [3], [4]. In future research it is important to acknowledge this difference and to carefully select the most appropriate physiological response system for the specific research question at hand. This holds for all kinds of psychophysiological research on emotion, like for example fear conditioning studies. In this type of studies, SCR is often interpreted as an index of conditioned *fear* rather than a measure of anticipatory *arousal*. Hence, ASR should be preferred in studies employing just a single physiological measure to index negative valence. Furthermore, our results confirm that vagally-mediated HRV is a promising biological marker of individual differences in affective responding, specifically related to ASR potentiation and thus a defensive state. Resting HRV is associated with the ability to differentiate between emotions, which may be considered as the hallmark of adaptive, flexible and healthy functioning [31], [32]. Future research on individual differences in emotional expression may benefit from taking resting HRV into account. This may provide valuable insights into the demarcation from adaptive to maladaptive responding.
